# Bereavement reduces neutrophil oxidative burst only in older adults: role of the HPA axis and immunesenescence

**DOI:** 10.1186/1742-4933-11-13

**Published:** 2014-08-29

**Authors:** Ana Vitlic, Riyad Khanfer, Janet M Lord, Douglas Carroll, Anna C Phillips

**Affiliations:** 1 School of Sport, Exercise and Rehabilitation Sciences, University of Birmingham, Birmingham, UK; 2 MRC-Arthritis Research UK Centre for Musculoskeletal Ageing Research, University of Birmingham, Birmingham, UK; 3 City Hospital Eye Accident and Emergency Department, Sandwell and West Birmingham Hospitals NHS Trust, Birmingham, UK; 4 School of Immunity and Infection, University of Birmingham, Birmingham, UK

**Keywords:** Bereavement, Neutrophil function, Cortisol, DHEAS, Social support

## Abstract

**Background:**

The effect of the chronic stress of bereavement on immunity is poorly understood. Previous studies have demonstrated negative effects on immunity in older adults, and those who report higher depressive symptoms. The aim of the present study was to compare the effect of bereavement on neutrophil function in healthy young and old adults, also assessing serum levels of the stress hormones, cortisol and dehydroepiandrosterone sulphate (DHEAS). 41 young (mean age 32 years) and 52 older adults (mean age 72 years), bereaved and non-bereaved, took part in the study. They completed questionnaires on socio-demographic and health behaviour characteristics, as well as psychosocial variables, and provided a blood sample for analysis of neutrophil function (phagocytosis and reactive oxygen species (ROS) production) and stress hormone analysis.

**Results:**

Bereaved participants in both age groups reported more symptoms of depression and anxiety than controls and scored moderately highly on bereavement-specific questionnaires for these symptoms. Despite this, young bereaved participants showed robust neutrophil function when compared to age-matched non-bereaved controls, and comparable stress hormone levels, while reduced neutrophil ROS production and raised stress hormone levels (cortisol:DHEAS ratio) were seen in the older bereaved group compared to their age-matched controls.

**Conclusions:**

Reduced neutrophil function among older bereaved participants may be the result of the inability to maintain stress hormone balance, specifically the cortisol:DHEAS ratio.

## Introduction

Bereavement is a stressful life event often accompanied by grief after the loss of someone close [1] and, as such, has numerous consequences for physical and mental health [2]. In addition to the increase in morbidity and mortality associated with bereavement in older adults [3-5], particularly in the case of the unexpected death [6], bereavement has been shown to have a number of adverse effects on immunity [7]. For example, bereavement in the year prior to vaccination related to lower antibody responses to two different influenza strains in older adults (mean age 75 years) [8], and decreased lymphocyte response to phytohaemagglutinin (PHA) [9]. On the molecular level, the expression of the genes specifically involved in B cell immunity was down-regulated in the bereaved older adults (aged 61–83 years) when compared to age- and sex-matched controls [10]. Among younger adults, bereaved parents aged 38–61 years experienced a decrease and increase in the number of regulatory and helper T cells, respectively, compared to matched controls after the sudden and unexpected death of their child [11]. In terms of the innate immune response, bereaved female spouses aged 57.1 ± 7.9 years (mean ± SD) had a poorer Natural Killer (NK) cell cytotoxic activity when compared to gender matched controls [12], and neutrophil reactive oxygen species (ROS) production was lower in bereaved older adults (mean age 72 years) when compared to the age- and sex-matched non-bereaved participants [13]. In contrast, a group of middle aged widows (mean age 56 years) showed preserved immune response compared to non-bereaved controls [14]. However, within the bereaved group, those with depressive symptoms had lower NK cell activity and response to mitogen stimulation than those without [14].

Previous studies of the impact of physical stress (e.g. hip fracture) have shown that impaired immune function, specifically neutrophil ROS production was only seen among older adults with concomitant immunosenescence and did not occur in young patients with a similar level of trauma [15]. Importantly in this study HPA axis activity, specifically a raised cortisol:DHEAS ratio was highest in those patients with the lowest neutrophil ROS production and also lower in patients who developed infection [15]. Further, a subsequent study revealed that reduced ROS production and a higher cortisol:DHEAS was observed in those hip fracture patients with depressive symptoms when compared to both those patients without depression and healthy age-matched controls [16]. These data suggest that the effects of some types of stress on immunity may only be observed among older adults, or among those with poorer psychological status, e.g., high depressive symptoms.

Stress activates the hypothalamo-pituitary-adrenal axis and subsequently induces the secretion of cortisol, a hormone with immune suppressive effects [17]. DHEAS, also secreted by the adrenal gland in response to stress, is considered to be immune-enhancing [18]. Whilst cortisol has been shown to decrease the adhesion and increase mobility of the neutrophils [19,20], DHEAS increased neutrophil ROS production *in vitro*[21]. An imbalance between these two hormones, i.e., a high cortisol:DHEAS ratio can arise in response to stress [22,23] and have negative implications for immunity including increased risk of bacterial infection [24], whereas in the young fracture patients the ratio remained low. Further, our previous research in older adults showed a higher cortisol:DHEAS ratio in bereaved participants when compared to age- and sex-matched controls [13]. Indeed, with ageing levels of DHEAS decline whereas cortisol continues to be produced, termed adrenopause [25], thus resulting in a higher cortisol:DHEAS ratio. Whether the same increased stress hormone ratio and associated reduction in neutrophil function would be observed in younger adults suffering the stress of bereavement is not known.

Consequently, the present study sought to extend our previous research which showed reduced neutrophil function in older bereaved adults [13]. Specifically, it compared neutrophil function and the cortisol:DHEAS ratio in four groups of participants: younger bereaved adults; non-bereaved young participants; older bereaved adults and non-bereaved age-matched controls.

## Results

### Demographic, health behaviour and psychosocial characteristics

Table 1 shows that bereaved participants and controls were reasonably well matched on most socio-demographic and health behaviour variables in both young and old groups, with the exception of occupational status (*p* = .02), and the medication (*p* = .04) in the young. Young bereaved were more likely to hold manual occupations, and to take medication, mainly anti-hypertensives and non-steroidal asthma treatments. The bereaved in both groups reported more symptoms of depression and anxiety than controls. Social support availability did not differ between groups in either of the age cohorts. Bereaved participants scored moderately highly on both the CBI and IES, albeit slightly lower than bereaved participants in previous research [26-29]. In the younger group, two bereaved participants had lost a spouse (9.5%), eight had lost a parent (38.1%), nine a grandparent (42.9%), and two a distant relative, e.g. parent-in-law (9.5%). For the older group, the respective values were 17 (65%), 3 (12%) and 6 (23). The death was expected in 86% of cases in the younger group, and in 84% in the older group.

**Table 1:** **Socio**-**demographic**, **health behaviour and psychosocial characteristics of bereaved and non**-**bereaved participants**

	**Young**			**Older**		
	**Bereaved (N = 21)**	**Non**-**bereaved (N = 20)**		**Bereaved (N = 26)**	**Non**-**bereaved (N = 26)**	
	**N (%) / Mean (SD)**		***p***	**N (%) / Mean (SD)**		***p***
Age (years)	31.8 (9.03)	31.7 (8.41)	.97	71.3 (5.79)	72.6 (5.72)	.42
Gender (Female)	9 (43)	10 (50)	.65	18 (69)	17 (65)	.77
Ethnicity (Caucasian)	21 (100)	17 (85)	.07	26 (100)	25 (96)	.31
Occupational status (non-manual)	16 (76)	20 (100)	.02	19 (76)	21 (81)	.68
Taking medications	4 (19)	0 (0)	.04	15 (60)	15 (58)	.87
Alcohol intake (daily or more)	5 (25)	2 (10)	.21	10 (38)	7 (27)	.38
Smokers	5 (25)	4 (20)	.71	2 (1)	3 (12)	.64
Body Mass Index	24.3 (4.20)	23.0 (2.70)	.27	26.2 (3.93)	25.5 (3.36)	.52
Exercise score	7.8 (5.95)	9.6 (4.83)	.30	5.5 (1.35)	8.0 (1.35)	.21
Fruit and vegetable consumption score	9.8 (2.96)	8.9 (2.27)	.28	9.3 (2.03)	9.9 (2.04)	.27
Fat consumption score	10.6 (3.53)	11.3 (2.65)	.49	10.6 (3.66)	10.0 (3.21)	.56
HADS anxiety score^a^	8.0 (4.63)	5.2 (3.08)	.03	8.6 (4.90)	4.2 (2.95)	<.001
HADS depression score^a^	4.7 (3.15)	2.5 (2.50)	.02	6.1 (5.54)	2.42 (2.32)	.003
Social support score (MOS)^a^	73.8 (17.68)	80.4 (12.65 )	.18	70.4 (19.61)	80.2 (16.26)	.06
Core bereavement items (CBI)	23.2 (10.93)	-	-	29.8 (13.28)	-	-
The Impact Event Scale (IES)	33.1 (15.52)	-	-	33.2 (15.6)	-	-
Death expected - yes	18 (86)	-	-	21 (84)	-	-
Bereavement type Spousal	2(10)	-	-	17 (65)	-	-
Close relative (parent)	8 (38)	-	-	3 (12)	-	-
Distant relative/friend	11 (52)	-	-	6 (23)	-	-

^a^presents significant difference between bereaved and controls.

### Immune and hormone data between young and older groups of participants

#### Neutrophil function

**Figure 1:**
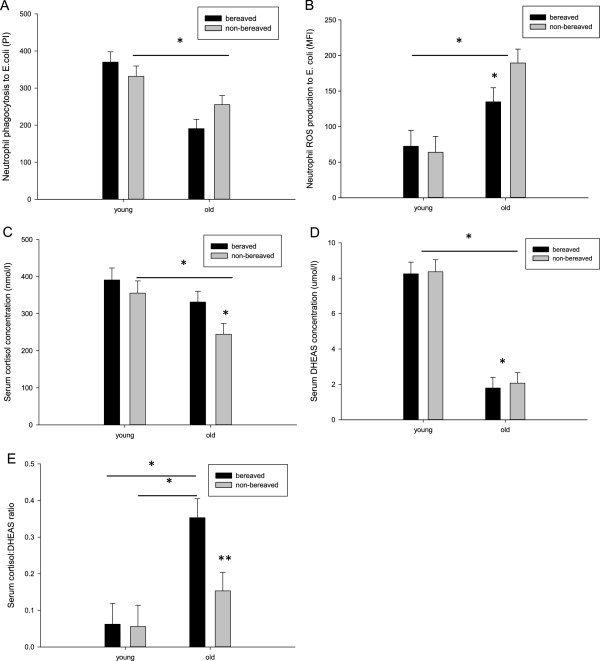
**Immune and hormone differences between bereaved and controls, old and young. (A)** Neutrophil phagocytosis of FITC labelled *E.coli* presented as phagocytic index (bacteria ingested (MFI) ×% neutrophils uptaking bacteria) between young and old, bereaved and non-bereaved subjects. **(B)** Neutrophil superoxide production in response to *E.coli* presented as mean fluorescence intensity (MFI), between young and old, bereaved and non-bereaved. Serum **(C)** cortisol, **(D)** DHEAS or **(E)** the cortisol:DHEAS ratio for young and old, bereaved and non-bereaved subjects. Error bars are SEM and * indicates *p* < .05, ** indicates p < 0.01.

For neutrophil phagocytosis, a 2x2 age group versus bereavement group ANOVA comparing neutrophil phagocytosis in young bereaved and matched controls with older bereaved and controls revealed the significant main effect of age, F(1,87) = 31.45, *p* < .001, η^2^ = .265, such that younger participants showed higher phagocytosis overall than older adults, but there was no overall main effect of bereavement, F(1,87) = 0.26, *p* = .61, η^2^ = .003, nor bereavement * age interaction effect, F(1,87) = 1.94, *p* = .17, η^2^ = .022 (Figure 1A). Repeated analyses with adjustment for occupational status and medication usage revealed the same significant main effect of age, *p* < .001.

For neutrophil ROS generation 2x2 ANOVA comparing neutrophil ROS production between young and old, bereaved and control revealed a significant main effect of age, F(1,87) = 34.4, *p* < .001, η^2^ = .284, such that older participants surprisingly had higher neutrophil superoxide burst than younger subjects. There was, however, no main effect of bereavement overall, F(1,87) = 1.02, *p* = .31, η^2^ = .012, nor bereavement * age interaction effect, F(1,87) = 2.63, *p* = .11, η^2^ = .029. Pairwise comparison revealed that the lack of the effect between bereaved subjects and controls was driven by the comparable ROS production in the younger group (*p* = .69), while there was a significant effect of bereavement in the older group (*p* = .05), such that older bereaved subjects had lower ROS production than older controls (Figure 1B). Repeated analysis with covariate adjustment also revealed a main effect of age (*p* < .001).

#### Serum stress hormone concentrations

For cortisol, 2x2 ANOVA between young and old bereaved and controls showed a significant main effect of age, F(1,84) = 8.80, *p* = .004, η^2^ = .095, such that younger participants had higher serum cortisol levels, but no main effect of bereavement, F(1,84) = 3.28, *p* = .07, η^2^ = .038, nor bereavement * age interaction effect, F(1,84) = 1.42, *p* = .24, η^2^ = .017. Pairwise comparison revealed a significant effect of bereavement in the older group (*p* = .03), such that older bereaved subjects had higher cortisol levels than controls, while there was no difference in the young (*p* = .68) (Figure 1C). Repeated analyses with covariates adjustment showed a similar main effect of age (*p* = .03).

For DHEAS, 2x2 ANOVA using young and old, bereaved and non-bereaved showed a significant main effect of age, F(1,84) =62.08, *p* < .001, η^2^ = .425, such that younger subjects had higher serum DHEAS, but no main effect of bereavement, F(1,84) = 1.95, *p* = .17, η^2^ = .023, nor bereavement * age interaction effect, F(1,84) = 1.77, *p* = .19, η^2^ = .021, was seen (Figure 1D). Pairwise comparison revealed a significant bereavement effect in the older group (*p* = .04), such that older bereaved had lower DHEAS than non-bereaved older controls, while the levels of this hormone were comparable between the groups in the young (*p* = .97). Repeated subsequent analyses including the covariates showed a similar main effect of age, *p* < .001.

For the cortisol:DHEAS ratio, 2x2 ANOVA revealed a significant main effect of age, F(1,84) = 14.35, *p* < .001, η^2^ = .146, and the trend towards an effect of bereavement, F(1,84) = 3.59, *p* = .06, η^2^ = .041, such that younger participants and control participants had a lower cortisol:DHEAS ratio, respectively. There was, however, no bereavement * age interaction effect, F(1,84) = 2.33, *p* = .13, η^2^ = .027. Pairwise comparison revealed that the trend towards a bereavement effect was driven by the differences in older group (*p* = .01), while the ratio was comparable between the young (*p* = .80) (Figure 1E). Covariate analyses confirmed a main effect of age, *p* = .002.

### Psychological factors and immune and hormone measures within the bereaved group

Correlations within the bereaved revealed no association between neutrophil function and any of the psychosocial and socio-demographic variables. There was also no significant difference in neutrophil function between those bereaved participants who had lost a spouse or parent and those who had lost a more distant relative. Correlation analysis within the bereaved group revealed that those with higher CBI scores, indicative of greater grief had a higher cortisol:DHEAS ratio, r(42) = .34, *p* = .03, and those who reported higher social support had lower cortisol:DHEAS ratio, r(42) = − .31, *p* = .04. When bereaved participants were separated into two groups based on who they had lost, there was a difference between the groups in their cortisol:DHEAS ratio, F(1,42) = 9.04, *p* = .004, η^2^ = .177, such that those who had lost someone more distant had a lower cortisol:DHEAS ratio.

## Discussion

In the present study, there was no difference in neutrophil function and serum hormone levels between bereaved and controls overall, with the main differences emerging between the two age groups. This was despite the differences in psychosocial variables that showed higher depressive and anxiety symptoms in the bereaved. Closer analyses revealed the younger group as responsible for these null findings, since neutrophil function and stress hormone levels were comparable between the bereaved and control groups in the young. On the other hand, older bereaved subjects had poorer ROS production, and a higher cortisol:DHEAS ratio when compared to the matched non-bereaved older adults, consistent with the previous studies of bereavement and immune function in older adults [8,13].

The present observational study did not include assessment of infection susceptibility or expose the bereaved participants to an immune challenge, thus the impact of these differences on immunity cannot be stated categorically. However, the cortisol:DHEAS ratio shows an almost two-fold increase in the older bereaved subjects. This may have physiological significance as such an increase is comparable to that previously reported in older adults after hip-fracture who developed post-trauma infection compared with patients who did not develop infection [24]. Moreover, although the less than two-fold lower neutrophil ROS production in the older bereaved group poses the question of clinical significance, we believe this is possible. For example, Butcher et al. in the same hip fracture study showed that a significantly lower superoxide production, even though less than two-fold lower, was observed in elderly hip fracture patients who later developed infection [24]. Therefore, it is possible that even small decrease in neutrophil superoxide production is large enough to weaken immune protection in stressed older adults and consequently lead to the development of infection. Further support for this contention can be found in the superoxide production to fMLP of isolated neutrophils that was significantly, but less than two-fold lower in patients with systemic sclerosis, an autoimmune disease in which patients are more susceptible to infections [30]. Taken together, these data suggest that in case of neutrophil function, both preserved phagocytosis and ROS production are necessary for the adequate protection of the individual.

The absence of an effect of bereavement on neutrophil function in the younger sample is perhaps surprising given the high levels of depression and anxiety symptoms among the bereaved, similar to those recorded for the older bereaved sample (Table 1). In addition, responses to questionnaires measuring grief, and impact of the bereavement indicated significant feelings of loss in the present study in both groups. However, only a limited number of studies have examined the effect of bereavement on immune function in younger adults. Lower numbers of regulatory T cell and helper T cells [11], and lower NK cell cytotoxicity [31] were reported for individuals who had experienced sudden/unexpected death of a close friend or family member. Further, no group differences in NK cell activity was observed between middle aged widows and married controls [14], although NK cell activity and the response to mitogens was poorer in a small sample of widows with symptoms of major depression. In the present study, although depressive symptomatology was higher among the bereaved, only one bereaved participant met the criteria for severe depression or higher (HADS ≥ 11). There are several potential explanations for the present null findings for neutrophil function in the young bereaved. It is possible that the intact neutrophil function was attributable to losses in the present study being of less close relationships than those of older adults, only 10% of the bereavements were spousal in the younger sample, the comparable figure for the older participants was 65% [13] (Table 1). However, there was no difference in neutrophil function in the present study between those who had lost a close relation (spouse, parent) and those who had lost a more distant relative (grandparent, parent-in-law). Further, social support is an unlikely explanation for preserved immunity in the present study as the support scores of the young bereaved were virtually identical to those found in older bereaved participants (Table 1), who showed reduced neutrophil ROS production.

The most plausible explanation for the preservation of neutrophil function in young but not older bereaved subjects we propose is the difference in the HPA axis response between the two groups, superimposed upon the aged neutrophil. Previous research indicates that stress may affect immune function more readily in the context of concomitant immune ageing. For example, lower secretory Immunoglobulin A, [32], and higher antibody titres against cytomegalovirus [33] were specifically characteristic of older caregivers. In general, there is consistent evidence of compromised immune function in older spousal caregivers for partners with dementia [34,35], whereas the results from the studies of younger caregivers are more variable [33,34] In that context, it was shown that the cortisol:DHEAS ratio was only raised in the older bereaved subjects compared to their controls and not in the younger bereaved group. With the well documented and opposing effects of cortisol [35] and DHEAS [15,36] on neutrophil ROS production, this proposal has biological validity.

The present study is not without limitations. First, the sample size can be regarded as small; however, bereaved participants are notoriously difficult to recruit and the sample size is comparable to that recruited to previous studies of immunity and bereavement [13,31]. Second, it could be argued that the preserved immune function in the present sample was due to bias such that those who are less stressed by or coping better with bereavement might be more likely to take part. However, the scores on CBI and IES suggested that the bereavements were significantly stressful.

## Conclusions

In conclusion, unlike older bereaved adults, younger bereaved participants showed no detrimental effect of bereavement on neutrophil function and stress hormone concentrations when compared to the matched non-bereaved controls. This is most likely attributable to the absence of immunosenescence and adrenopause in this younger aged bereaved group.

## Methods

### Participants

21 young bereaved adults and 20 age- and sex-matched non-bereaved controls, as well as 26 older bereaved adults and 26 controls participated in the study. Recruitment was conducted mainly via local advertisements and the Bereavement Care Centre, Queen Elizabeth Hospital, Birmingham. The bereaved group comprised participants who suffered bereavement in the past two months. None of the participants suffered from a chronic immune disorder or acute infection, and none were taking immunosuppressive medication.

### Study design and procedure

Participants attended a morning testing session where they completed a questionnaire pack and provided a blood sample. Informed written consent was obtained, and the study was approved by the local Ethics Committee.

### Questionnaires

Groups were compared on general socio-demographic variables, as well as health behaviours. The latter were assessed using an adaptation of the Whitehall II study questionnaire [36]. The Hospital Anxiety and Depression Scale (HADS) [37], was used to determine depression and anxiety symptoms in all participants, and the Cronbach’s alpha in the present study was .86 for anxiety and .80 for depression. The availability of social support was examined using the Medical Outcomes Study (MOS) Social Support Survey [38]. The Cronbach’s alpha in the current sample was .96.

Bereaved participants were asked about their recent bereavement using the Core Bereavement Items questionnaire (CBI, [27]), and the Impact of Event Scale (IES, [28]). The CBI assesses the feelings of the bereaved on a 4-point scale from 0 - *never*, to 3 - *continuously* happening. An example of a typical item is ‘Do reminders of this person such as photos, situations etc. cause you to feel loneliness’. Previously used in bereavement research [39,40], the scale showed good internal consistency at .91; and .94 in the present study. The IES asks about frequency of feelings about the bereavement (e.g. how frequently ‘you had dreams about it’), with higher scores meaning higher negative impact. The scale shows good internal consistency (.79-.92) [29]; and .89 in the current sample. They were also asked who the deceased person was in relation to them, and whether the death was expected or not.

### Blood sampling and assays

Venous blood was collected, in one heparin, for neutrophil functional assessment, and one plain tube for serum hormone analyses. Serum from the plain tube was stored at −20°C for future ELISA analysis (IBL international, Hamburg, Germany). Neutrophil phagocytosis and oxidative burst activity were assessed using two commercial kits (Phagotest and Bursttest, respectively, Orpegen Pharma GmvH, Heilderberg, Germany), following the suppliers protocol. Phagocytic ability was presented as phagocytic index which was calculated as% phagocytic neutrophils x MFI, where MFI is mean fluorescence intensity measured by flow cytometer. The difference between MFI in the test sample (with E.coli) and control sample (with wash buffer) was used to measure the oxidative burst activity of neutrophils.

### Statistical analyses

Comparison between the bereaved and non-bereaved on socio-demographics, and questionnaire scores were conducted by ANOVA and chi-square as appropriate; with effect sizes reported as η^2^. Further, 2x2 bereavement group * age group ANOVAs were used to compare immune and hormone measures in the young and old, bereaved and controls. Neutrophil function and hormone levels were skewed and therefore subjected to log transformation. Significantly different demographic or health behaviour variables between groups were controlled for in further ANCOVAs. Correlations were used within younger bereaved group only to examine whether the cortisol:DHEAS ratio or any other questionnaire variables were related to neutrophil function. Further, bereaved participants were divided into two groups (those who lost spouse or parent versus those who lost more distant relative), and differences between them on neutrophil function and hormone status were examined using ANOVAs.

## Abbreviations

DHEAS: Dehydroepiandrosterone sulphate; ROS: Reactive oxygen species; PHA: Phytohaemagglutinin; NK: Natural killer; SD: Standard deviation; CBI: Core bereavement item; IES: Impact of event scale; PI: Phagocytic index; MFI: Mean fluorescence intensity; HADS: Hospital anxiety and depression scale; MOS: Medical outcome survey; ANOVA: Analysis of variance; ANCOVA: Analysis of covariance; SEM: Standard error of the mean.

## Competing interests

The authors declare that they have no competing interests.

## Authors’ contributions

AV participated in the collection of data, carried out the immunoassays and hormone ELISAs, performed the statistical analysis and drafted the manuscript; RK participated in the collection of data, immunoassays and ELISAs; JML participated in the design of the study and helped with the draft of the manuscript, as well as with hormone ELISAs; DC - participated with the design of the study and helped with the draft of the manuscript and statistical analysis; ACP - participated with the design of the study, and performed the statistical analysis and helped with the draft of the manuscript. All authors read and approved the final manuscript.
